# A chitin deacetylase from the endophytic fungus *Pestalotiopsis* sp. efficiently inactivates the elicitor activity of chitin oligomers in rice cells

**DOI:** 10.1038/srep38018

**Published:** 2016-11-30

**Authors:** Stefan Cord-Landwehr, Rebecca L. J. Melcher, Stephan Kolkenbrock, Bruno M. Moerschbacher

**Affiliations:** 1 Institut für Biologie und Biotechnologie der Pflanzen, Westfälische Wilhelms-Universität Münster, Schlossplatz 8, 48143 Münster, Germany

## Abstract

To successfully survive in plants, endophytes need strategies to avoid being detected by the plant immune system, as the cell walls of endophytes contain easily detectible chitin. It is possible that endophytes “hide” this chitin from the plant immune system by modifying it, or oligomers derived from it, using chitin deacetylases (CDA). To explore this hypothesis, we identified and expressed a CDA from *Pestalotiopsis* sp. (PesCDA), an endophytic fungus, in *E. coli* and characterized this enzyme and its chitosan oligomer products. We found that when PesCDA modifies chitin oligomers, the products are partially deacetylated chitosan oligomers with a specific acetylation pattern: GlcNAc-GlcNAc-(GlcN)_n_-GlcNAc (n ≥ 1). Then, in a bioactivity assay where suspension-cultured rice cells were incubated with the PesCDA products (processed chitin hexamers), we found that, unlike the substrate hexamers, chitosan oligomer products no longer elicited the plant immune system. Thus, this endophytic enzyme can prevent the endophyte from being recognized by the plant immune system; this might represent a more general hypothesis for how certain fungi are able to live in or on their hosts.

The immune system of plants has evolved to defend them against pathogens, based on a sophisticated non-self surveillance system tuned to foreign molecules such as chitin, an evolutionary ancient^1^, tell-tale polymer present e.g. in fungi and insects, but absent in healthy plants. In plants, effective immune molecules are chitinases, because as they break down chitin, they weaken fungal cell walls and produce elicitor-active chitin oligomers^2^. These chitin oligomers are recognized by chitin-specific receptors, triggering further resistance reactions^3^,^4^,^5^,^6^.

This type of detection by the plant immune system has to be prevented by chitin-containing organisms that need to live on or in plants to survive. Such organisms include plant pathogens, in particular biotrophic pathogens, as well as endophytic fungi, which live inside the tissues of their host plants without causing visible symptoms, and without an apparent beneficial role for the plant host^7^.

Previous studies have hypothesized that these organisms, specifically fungi, prevent chitin from being recognized by the plant immune system in two ways^2^,^8^: First, fungi might mask the chitin in their cell wall, e.g. by covering it with other polymers, or by deacetylating it into chitosan. Chitosan is the fully or partially deacetylated derivative of chitin naturally found in the cell wall of Zygomycetes fungi and in intercellularly growing mycelia of some pathogenic fungi^9^,^10^. Chitosan in the cell wall is thought to be synthesized as a chitin derivative, such that once chitin is made, it is immediately deacetylated to chitosan^11^. Second, it has been hypothesised that fungi can also modify the elicitor-active chitin oligomers that are produced by the chitinases involved in the plants’ immune response. Here, these chitin oligomers may be inactivated by being bound, degraded, or deacetylated^2^,^10^. For both hypotheses, deacetylation may be the most likely inactivator of chitin, as it is known that fully deacetylated chitosan oligomers are not bound by plants’ receptors and therefore do not lead to an immune response^5^,^12^.

In fungi, the key enzymes that convert chitin to chitosan are chitin deacetylases (CDA). CDAs can be found bound to the cell wall, where they may be directly involved in the production of the cell wall chitosan (as in the first hypothesis mentioned above), but they are also secreted by many organisms^8^,^11^. Secreted, soluble CDAs have been assumed to be responsible for the deacetylation of chitin oligomers produced by the plant immune response (mentioned in the second hypothesis), thus preventing any resistance reactions from being induced^13^.

While various previous studies have investigated CDAs from the plant pathogenic fungi *Colletotrichum lindemuthianum* (ColLinCDA)^14^ or *Puccinia graminis* f. sp. *tritici* (PgtCDA)^15^ — in which the CDA has been purified from culture media, heterologously expressed in *E. coli* and/or *Pichia pastoris*, and/or examined for its crystal structure and mode of action^13^,^14^,^15^,^16^,^17^— no previous studies actually confirmed the biological role of this enzyme or analysed the bioactivity of its produced chitosan oligomers. Therefore, the roles of fungal CDAs in evading the plant immune system, either by cell wall modification or chitin oligomer deacetylation, have not been proven experimentally.

To learn more about the biological role of CDAs regarding the hypothesis that they help to evade the plant immune system by modifying chitin oligomers, we characterized a soluble CDA from the endophytic fungus *Pestalotiopsis* sp., which lives in the leaves of tropical trees of the Western Ghats (India), and its products. The products of chitin oligomer deacetylation were analysed revealing that the enzyme produces chitosan oligomers with a novel pattern of acetylation (PA), namely GlcNAc-GlcNAc-(GlcN)_n_-GlcNAc (n ≥ 1). Bioactivity assays with rice cells and the PesCDA products showed that the enzyme is able to convert elicitor-active compounds into elicitor-inactive ones, and therefore might help the fungus to grow in the plants (Fig. 1).

## Results

From the endophyte *Pestalotiopsis* sp., we identified a CDA that was produced by the organism when chitosan was present in the cultivation medium. As such, we were able to observe how this organism might handle the chitin related plant immune system by characterizing the CDA enzyme and exploring the interaction of its produced chitosan oligomers with plant cells.

### *pescda* gene identification, cloning and expression

**Figure 2:**
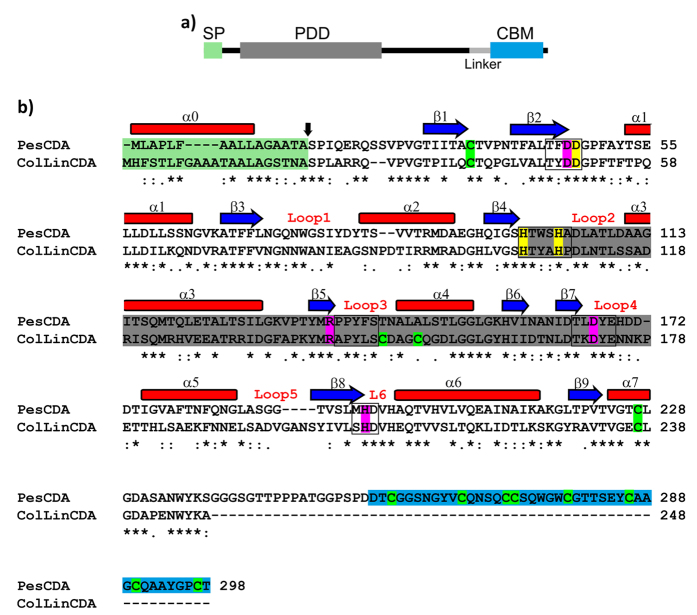
Domain architecture of the chitin deacetylase PesCDA from *Pestalotiopsis sp*. according to Pfam^44^ and Signal P^45^ predictions (**a**). The enzyme contains an N-terminal putative signal peptide (SP), a polysaccharide deacetylase domain (PDD), and a C-terminal carbohydrate binding module representing motif family 18 (CBM18)^56^. ClustalW amino acid sequence alignment of PesCDA and ColLinCDA^57^ (**b**). The catalytically active amino acids are labelled in magenta, the cofactor-binding site in yellow, the cysteine residues in green and the five conserved motifs (MT1-5) of carbohydrate esterase family 4 (CE-4) are indicated by black boxes^23^. The six loops were identified according to the crystal structure of *Vibrio cholerae* CDA^24^. SignalP^45^ was used to predict the cleavage site of the putative signal peptide (black arrow). Blue arrows indicate the predicted β-sheets and the red boxes α-helices in the CDAs using PSIPRED^46^, labelled according to the secondary structure of CE-4^23^.

We identified the gene (GenBank accession number: KY024221) in *Pestalotiopsis* sp. encoding a CDA using CODEHOP-PCR with primers based on a multiple sequence alignment of fungal CDA genes and 3′–5′-RACE-PCR. The predicted molecular mass of the enzyme is 30.9 kDa and the protein sequence reveals two domains separated by a linker region: an N-terminal polysaccharide deacetylase domain (PDD) containing the active site of the enzyme and a C-terminal chitin-binding module (CBM). The catalytic PDD found in PesCDA contains the seven conserved α-helices, eight conserved β-sheets, and all five conserved motifs that form the active site of the enzyme and are part of all described CDAs. Additionally, the PDD of PesCDA includes the CDA-typical metal-binding and catalytically active amino acids. Cysteine residues, one each in the N-terminal and C-terminal parts of the domain, occupy the same positions as found in ColLinCDA, where they are known to form a disulfide bond^13^. The protein also possesses a putative N-terminal signal peptide (Fig. 2).

To characterise the enzyme and analyse the function of its products, we first had to produce it in *E. coli* Rosetta 2 (DE3) cells as maltose binding fusion protein and to purify it by Strep-tactin affinity chromatography. Analysis of the isolated protein using SDS-PAGE followed by Coomassie Brilliant Blue staining and western blotting revealed that the construct yields an intact polypeptide of the anticipated length, although additional bands were also detected (Supplementary Figure S1).

### Characterisation of the enzyme

**Figure 3:**
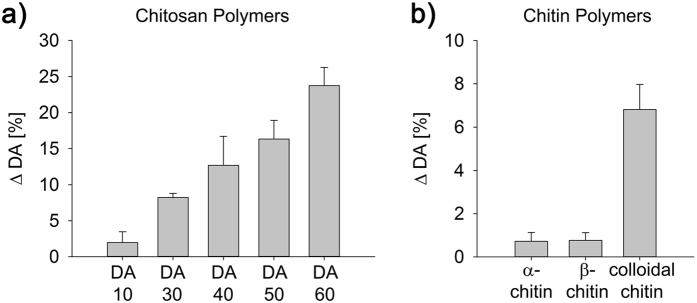
Activity of PesCDA against chitosan (**a**) and chitin (**b**) polymers. The substrates (1 mg/ml) were incubated for 24 h at 37 °C in 50 mM TEA (pH 7.0) with PesCDA (4 μg/ml). The activity of the enzyme (ΔDA) was calculated based on the amount of released acetate. The standard deviation was calculated based on three independent experiments.

Biochemical studies including analysis of substrate specificity were performed with PesCDA to identify its basic characteristic and to be able to compare it with well-studied CDAs, in particular ColLinCDA from *C. lindemuthianum*. We found that PesCDA is active as a chitin deacetylase when tested on fully acetylated chitin oligomers, with the tetramer being the smallest productive substrate (Supplementary Figure S2). We used chitin pentamers as a substrate to determine the optimal reaction conditions, revealing a pH optimum of 8.0 and a temperature optimum of 55 °C (Supplementary Figure S3 and Supplementary Figure S4). PesCDA was pre-incubated with 1 mM solutions of different cations as potential CDA activators (Ca^2+^, Fe^2+^, Mg^2+^, Mn^2+^ and Zn^2+^, presented as chloride salts) or with EDTA as a potential inhibitor. Most of these treatments do not affect enzyme activity, but FeCl_2_ causes almost complete inactivation, whereas MnCl_2_ reduces the activity of PesCDA by ~50% (Supplementary Table S1). The incubation of PesCDA with chitin pentamers in the presence of 100 mM ammonium acetate reduces enzyme activity by 20% (Supplementary Table S2). PesCDA was then tested for its activity against chitosan polymers that had degrees of acetylation (DA) of 10–60% as well as against colloidal chitin, α-chitin and β-chitin. The enzyme is inactive against insoluble α-chitin and β-chitin, slightly active against colloidal chitin, and most active against soluble chitosan polymers (Fig. 3). The activity of PesCDA increases with the DA of the substrate.

### Enzyme activity on chitin oligomers and product analysis

Analysing the bioactivity of the chitosan oligomers produced by this enzyme required a detailed analysis of their architecture; namely, we had to determine the amounts of the GlcNAc and GlcN units and their order. Matrix assisted laser desorption ionization – time of flight – mass spectrometry (MALDI-TOF-MS) analysis of chitin oligomers (GlcNAc_n_, with n varying from 2 to 6) incubated with PesCDA revealed that the enzyme is able to deacetylate chitin oligosaccharides larger than the trimer, yielding products with the formula GlcNAc_3_GlcN_(n-3)_ (Supplementary Figure S2). The quantity of intermediate products with more than three remaining GlcNAc units is higher for larger oligomers than for smaller ones after the same incubation period at the same substrate concentration. Longer incubations reduces the abundance of these intermediate products.

**Figure 4:**
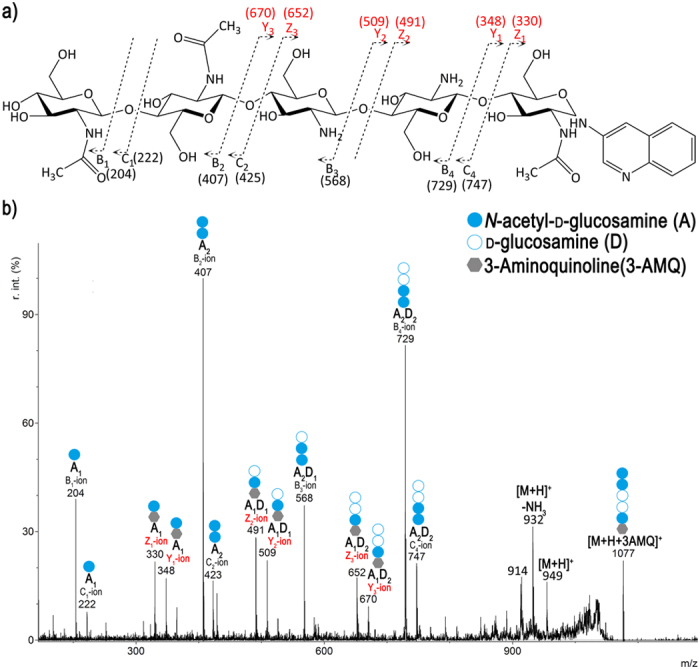
Chemical structure (**a**) and MALDI-TOF-MS/MS fragmentation pattern (**b**) of the double-deacetylated chitin pentamer (A_3_D_2_-AADDA) with 3-Aminoquinoline (3-AMQ) on the reducing end as an H^+^-adduct. This A_3_D_2_ was produced by incubation of chitin hexamer (A_6_) (1 mg/ml) with PesCDA (4 μg/ml) under the standard conditions. Nomenclature of the fragments is according to Domon and Costello^18^; fragments derived from the reducing end (Y- and Z-ions) are marked in red letters.

MALDI-TOF-MS/MS was then used to analyse the PesCDA products at different time points during the incubation (2, 7, and 24 h) to determine their pattern of acetylation. The main product for each substrate after incubation for 2 h is the mono-deacetylated derivative, and further products only appear after longer incubation times. Careful analysis of the fragments of partially deacetylated oligomers produced by MALDI-TOF-MS and MALDI-TOF-MS/MS allows to decipher the sequence of the GlcNAc (A) and GlcN (D) units of these chitosan oligomers. MALDI-TOF-MS/MS and fragmentation pattern analysis are shown for the double-deacetylated pentamer product A_3_D_2_ as an example (*m/z* = 1076.39 with 3-AMQ and H^+^) in Fig. 4. The nomenclature of Domon and Costello for carbohydrate fragmentation in MS is used^18^, and the cleavage site for each fragment in the MS/MS spectrum is shown in Fig. 4a. In this manner, we were able to identify the sequence AADDA for A_3_D_2_ (Fig. 4b); the same approach was applied to the deacetylation products of A_4_ (A_3_D_1_), A_5_ (A_4_D_1_ and A_3_D_2_) and A_6_ (A_5_D_1_, A_4_D_2_ and A_3_D_3_) (Supplementary Figure S5 - Supplementary Figure S9).

**Figure 5:**
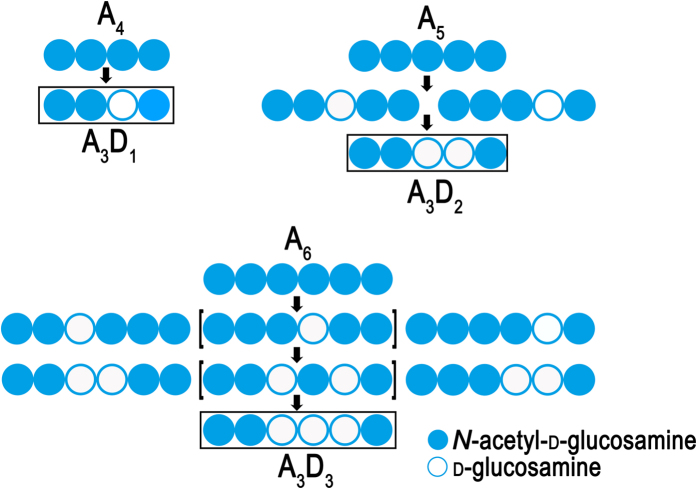
Mode of action of PesCDA against chitin tetramer (A_4_), pentamer (A_5_) and hexamer (A_6_) substrates. Due to the limitations of MALDI-TOF-MS/MS analysis, it was not possible to determine unequivocally the presence of the two hexamer products given in square brackets. The boxed patterns are end products of the reactions.

The mode of PesCDA action, as described by the pattern of the intermediate and end products it produces, is shown in Fig. 5. These data clearly show that the enzyme can deacetylate all GlcNAc units in the chitin oligosaccharides between the reducing end unit and the penultimate unit of the non-reducing end. Thus, starting from a pure chitin oligosaccharide with a specific DP, the enzyme produces not only chitosan oligosaccharides with a specific DA, but also with a specific PA, described as: GlcNAc_n_→ GlcNAc-GlcNAc-(GlcN)_n-3_-GlcNAc.

### Elicitor activity of oligomeric PesCDA products

**Figure 6:**
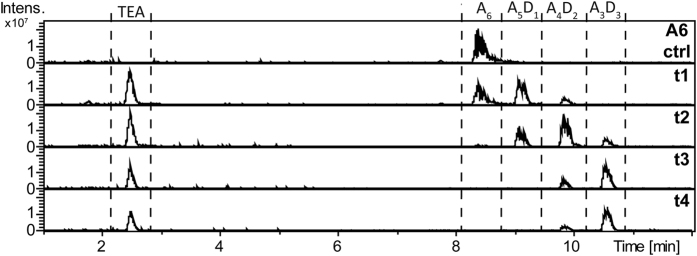
HILIC-ESI-MS base peak chromatogram of the different PesCDA derived paCOS solutions used for the oxidative burst experiments in *Oryza sativa* cell suspension cultures. Chitin hexamer (A_6_) (1 mg/ml) was incubated under standard conditions with PesCDA (4 μg/ml) for 2 (t1), 7 (t2), 24 (t3), and 48 h (t4), and the reactions were stopped by removing the enzyme with 3 K PES filter.

**Figure 7:**
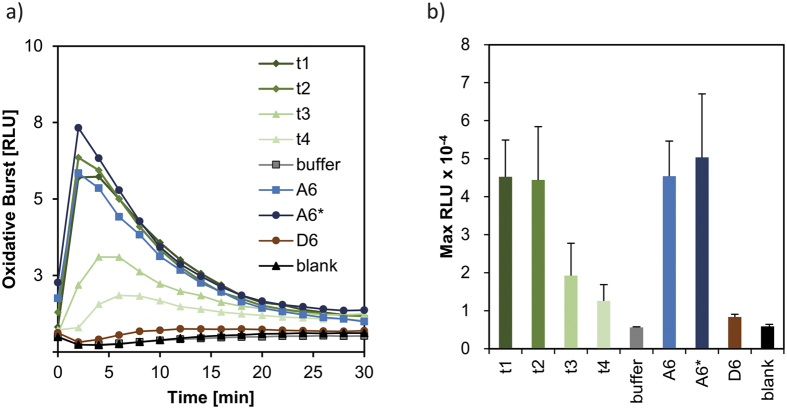
Oxidative burst in *Oryza sativa* cell suspension culture. ROS production was monitored over time after elicitation with products of chitin hexamer (A6) PesCDA incubation (**a**). Mean of maximal ROS production; the standard deviation was calculated based on three independent experiments (**b**). t1 to t4 refer to different time points of enzyme incubation (see Table 1); A6* was incubated with heat-deactivated enzyme; D6 chitosan hexamer.

The role of the CDA in the plant immune system’s interaction with chitin oligomers was investigated by using rice (*Oryza sativa*) cells, an established system with well-known receptors for chitin recognition^19^. Therefore, the chitin hexamer, a known elicitor in rice, was incubated with PesCDA. Table 1 and Fig. 6 show the results of the structural analyses of the end product, a triple-deacetylated chitosan with the pattern AADDDA, as well as of the intermediate products of the reaction. To assess the plant immune response, we used a rice cell suspension culture assay to detect the elicitation of an oxidative burst, i.e. the rapid and transient production of reactive oxygen species (ROS) over time via chemiluminescence (Fig. 7a and b) induced by the partially deacetylated chitosan oligosaccharides (paCOS). As control, we used the fully acetylated chitin hexamer GlcNAc_6_ and the fully deacetylated chitosan hexamer GlcN_6_. As expected, the fully acetylated chitin hexamer elicits an oxidative burst, whereas the fully deacetylated chitosan hexamer does not. Intermediate products of the early time points of incubation (2 and 7 h), mostly containing the mono- and double-deacetylated products A_5_D_1_ and A_4_D_2_, are elicitor active, while the end product A_3_D_3_ (AADDDA) dominating the samples from the late time points of incubation (24 and 48 h) is inactive. All elicitor-active intermediate products induce a strong oxidative burst within the first five minutes after elicitation.

## Discussion

To our knowledge, this is the first analysis of a chitin deacetylase from an endophytic organism that has revealed both its substrate specificity as well as the chemical structure and biological activity of its products.

Until now, as far as we know, no studies have determined how CDA enzymes and especially their products, partially deacetylated chitosans, are involved in the interaction between fungi and the plant immune system. Heterologous expression of fungal CDA genes in *E. coli* and detailed characterization of their products, although successful in rare cases^17^,^20^,^21^, has proven difficult. We overcame this obstacle by expressing the PesCDA as a fusion protein with MBP, which greatly increases the amount of active enzyme compared to when MBP is not fused, probably by increasing its solubility^22^.

Based on both the structural comparison and biochemical characterization, we found that PesCDA is similar to the previously described CDAs. The catalytically active PDD of PesCDA has the typical secondary structure elements (α-helices and β-sheets) and the five conserved motifs, including the catalytically active amino acids found in all chitin deacetylases and the six loops surrounding the active site of the enzyme^23^,^24^. The temperature and pH optima of PesCDA (55 °C and pH 8) are similar to those of other fungal CDA^25^. The ability of PesCDA to tolerate EDTA and the absence of activation effects in the presence of divalent cations indicate that the cofactor is tightly bound to the active site. The addition of 100 mM acetate reduces the enzyme activity by only 20%. In contrast to many other fungal CDA that are active against chitin oligomers including dimers^26^,^27^,^28^,^29^,^30^, PesCDA is only active against chitin oligomers larger than the trimer.

PesCDA is active on chitin oligomers with a DP > 3. During PesCDA’s first attack, these oligomers can be deacetylated at different positions among the units between the reducing end unit and the penultimate unit from the non-reducing end. Interestingly, when a chitin tetramer is used as the substrate, the first position of the deacetylation is similar for pathogen and endophyte derived enzymes. The PesCDA’s primary attack site on the chitin tetramer is similar to that of ColLinCDA and PgtCDA, both CDAs from plant pathogenic fungi, but PesCDA stops after the first deacetylation whereas PgtCDA in addition removes the acetyl group from the reducing end, and ColLinCDA continues to remove acetyl groups from the entire tetramer^15^,^16^. For PesCDA as well as for ColLinCDA, the deacetylation of the oligomers can be assumed to involve a multiple-chain-type mechanism, as shown by the predominance of mono-deacetylated oligomers during the early stages of incubation whereas the end products were generated only after longer incubation periods^8^,^21^,^29^. The interesting observation that the three above mentioned, rather closely related fungal CDA studied in detail so far yield different end products implies that they can be used as powerful tools for the production of fully defined paCOS. These are required for biotechnological investigations into structure-function relationships of partially acetylated chitosans, e.g. for agricultural, but also for biomedical and other applications^31^,^32^,^33^.

In contrast to ColLinCDA, which produces fully deacetylated chitosan oligomers as its end products, PesCDA yields partially acetylated chitosan oligomers as end products^16^. While it is known that fully deacetylated chitosan oligomers are elicitor inactive, because they are not recognized by the chitin receptors^12^,^34^, we were curious how the PesCDA products would behave. We wanted to find out how the specific deacetylation process of PesCDA influences the bioactivity of the generated partially acetylated chitosan oligomers. The fact that rice cells do not produce reactive oxygen species in the presence of hexameric PesCDA-processed chitin indicates that PesCDA functions by modifying an epitope that is recognized by the plant immune response. Given that the human immune system also recognises chitin oligomers^35^, that human pathogenic fungi such as *Cryptococcus neoformans* are known to partially deacetylate the chitin in their cell walls to chitosan^36^, and that the cell wall chitosan is required for *C. neoformans* virulence^37^, it is tempting to speculate that this CDA-based strategy of preventing induction of defence reactions is a more generic strategy.

The results of the bioactivity tests additionally support the hypothesis of Kaku and Shibuya, that chitin/chitosan oligomers must have at least five acetyl groups to enable binding and dimerization of the chitin receptors in rice^19^,^34^. Consequently, the presence of chitosan oligomers with at least five acetyl groups, the intermediate products of PesCDA, also result in the production of reactive oxygen species, triggering the plant immune system.

Our results show that PesCDA deacetylates chitin oligomers enough so that this endophyte can evade the host plant’s immune system. Interestingly, the organism found in leaves of tropical trees of the Western Ghats (India) produces a surprisingly large arsenal of chitin modifying enzymes including the analysed CDA^38^. The conspicuous multiplicity of chitin and chitosan modifying enzymes in this and other endophytic fungi suggests that they not only help the fungi evade the plant’s immune system, but may actually do other things, like help plants resist certain stresses. Therefore, studying other fungal CDAs on the one hand and further chitin modifying enzymes from *Pestalotiopsis* sp. on the other hand using the methods outlined in our study may help to determine other important functions of CDAs and chitin modifying enzymes that go beyond immune system evasion.

With this study, recombinant CDA with known regio-selectivities yielding chitosan oligomers with defined patterns of acetylation emerge as a powerful tool for the biotechnological production of potentially highly bio-active products. At slightly acidic pH values, GlcN units in paCOS are cationic, while GlcNAc units are hydrophobic. This allows paCOS to interact easily with their similarly partly anionic, partly hydrophobic target structures, such as proteins, nucleic acids, or phospholipid membranes. Thus, we can expect that the pattern of acetylation crucially determines molecular binding of paCOS to their targets and, thus, their bioactivities. The widespread occurrence of CDA not only in fungi, and in particular in pathogenic and endophytic fungi which often contain large CDA gene families^39^, but also e.g. in bacteria and insects, invites the question of their physiological roles. It is tempting to speculate that the role of soluble CDA is to produce specific chitosan oligomers with specific bioactivities.

## Materials and Methods

### Isolation of DNA from *Pestalotiopsis* sp.

A wild-type isolate of *Pestalotiopsis* sp^38^. was cultivated in a 250-ml flasks containing 50 ml of potato dextrose broth supplemented with 150 μg/l chloramphenicol, shaking at 120 rpm and 26 °C. The mycelium was separated from the medium by vacuum filtration through a 50-μm filter. DNA was isolated as previously described^40^.

### RNA isolation and cDNA synthesis

The fungus was cultivated as above for 5 d and 0.005% (w/v) colloidal chitin was added 24 h before harvesting. The mycelium was separated from the medium by vacuum filtration through a 50-μm filter. RNA isolation and cDNA synthesis were done as described previously^41^, using a modified form of the original method^42^.

### Identification and sequencing of the novel CDA gene

Consensus-degenerate hybrid oligonucleotide primer (CODEHOP) PCR^43^ was used to isolate CDA genes from the *Pestalotiopsis* sp. genome. The primers PDD_fw (5′-GTT GCC CTC ACC TTC GAY GAY GGN CC-3′) and PDD_bw (5′-GGA GCC GTA AGG AGG GCK CAT RTA NKT-3′) were designed based on a multiple alignment of several polysaccharide deacetylase domains (PDDs) from five fungal CDAs (*Podospora anserina*: CAP6016; *Blumeria graminis*: AAK84438; *Colletotrichum lindemuthianum*: AAT68493; *Aspergillus nidulans* FGSC A4: XP 659456; and *Emericella nidulans*: ACF22101). Genomic DNA and cDNA were used as templates for touchdown PCR, and the resulting products were sequenced and analysed using BLASTn. The cDNA for 3′–5′-RACE PCR was produced using the SMART^TM^ PCR cDNA Synthesis Kit from Clontech (Clontech, Mountain View, CA, USA) to isolate the complete genes corresponding to the sequences related to PDD-like sequences. Proof-reading PCR was then used to verify the nucleotide sequences.

### Bioinformatic analysis of PesCDA

The full-length amino acid sequence of the PesCDA was characterised by screening Pfam^44^, and analysing the sequence with SignalP v4.0^45^, DISOPRED2 (http://bioinf.cs.ucl.ac.uk/disopred) and PSIPRED (http://bioinf.cs.ucl.ac.uk/psipred)^46^.

### Construction of pET22b vectors containing the *pescda* cassette

The *pescda* cassette was amplified using the cDNA template and primers CDA-Nde_fw (5′-ATG CTC GCT CCC CTA TTC G-3′) and CDA-SalI_bw (5′-TTA AGT GCA GGG ACC GTA GG-3′) to add NdeI and SalI restriction sites for subcloning into vector pET22b(+)^47^ upstream of the strepII tag, simultaneously removing the *pel*B leader sequence. The *mbp* sequence was amplified from pMal-c2x (NEB, Ipswich, MA, USA) using primers malE_fw (5′-GCT CTA GAA ATA ATT TTG TTT AAG AAG GAG ATA TAA TTA TGA AAA-3′) and malE_bw (5′-GGA ATT CCA TAT GCC TTC CCT CGA TCC CG-3′) and was inserted upstream of the *pescda* cassette using XbaI and NdeI. The upstream *strepII tag* sequence was introduced by rolling-circle PCR with the 5′ phosphorylated primer pair RC-NStMBP_fw (5′-TTT GAA AAA GGC CCG ATG AAA ATC GAA GAA GGT AAA C-3′) and RC-NStMBP_bw (5′-CTG CGG ATG GCT CCA CATG ATTA TATC TCCT TCTT AAAC AAAA TTAT TC-3′), to produce the vector pET22b::NSt-MBP-CDA-CSt.

### Cultivation of recombinant bacteria

*E. coli* Rosetta2 (DE3) [pLysSRARE2] [pET22b::NSt-MBP-CDA-CSt] was incubated in 1 l of auto-induction medium (AIM) in a 2-l flask for 48 h at 26 °C, shaking at 120 rpm^48^. Cells were harvested by centrifugation (30 min, 4000 × *g*, 4 °C), re-suspended in 25 ml of fast protein liquid chromatography (FPLC) washing buffer (20 mM TEA, 400 mM NaCl, pH 8.0), and stored at −20 °C.

### Purification of PesCDA by FPLC

Cells were lysed by incubating at room temperature for 1 h and sonicating four times for 15 s at 20% amplitude using a Branson Digital Sonifier Model 250-D (Emerson, Ferguson, MO, USA). After centrifugation (30 min, 40,000 × *g*, 4 °C), the proteins were recovered from the supernatant by Strep-Tactin affinity chromatography (*Strep*-Tactin^®^ Superflow Plus Cartridge, Qiagen Hilden, Germany). The column was washed with 20 mM TEA containing 400 mM NaCl (pH 8.0), and proteins were eluted using 2.5 mM D-desthiobiotin in the same buffer. Purified enzymes were re-buffered in 20 mM TEA (pH 8.0) and stored at 4 °C in 10% (v/v) glycerol^47^.

### SDS-PAGE and western blotting

The concentration of protein in the crude and purified extracts was determined using the Bradford method^49^. We then boiled the samples for 10 min in reducing loading dye (62.5 mM Tris/HCl pH 6.8, 2% SDS, 5% glycerol, 0.04% bromophenol blue, 1 mM DTT), and loaded 3 μg of the purified recombinant protein or 15 μg of the crude extract onto 12% acrylamide gels for separation by SDS-PAGE^50^. Gels were stained with 0.05% Coomassie Brilliant Blue G-250 in 40% methanol plus 5% acetic acid, and proteins were transferred to nitrocellulose membranes for analysis by western blot using Strep-Tactin-HRP horseradish peroxidase with chemiluminescent substrate according to the manufacturer’s instructions (IBA, Göttingen, Germany).

### Acetate assay to determine enzyme activity and substrate DA after deacetylation

Enzyme activity was determined by measuring the amount of released acetate using the acetate assay kit (r-Biopharm, Darmstadt, Germany) modified for compatibility with a microtiter plate format. The adapted volumes were: 40 μl sample, 123 μl water, 100 μl solution 1, 20 μl solution 2, 20 μl solution 3 diluted 1:20, and 20 μl suspension 4 diluted 1:10, giving a total volume of 323 μl. The ΔE value of the samples was calculated using the equation given in the acetate assay kit. A standard curve generated using 0.05, 0.03, 0.015 and 0.003 g/l acetate was used to calculate the acetate concentration of the sample in g/l. This concentration was used to calculate the DA of the products after incubation with the enzyme, and the ΔDA, using equations 1 and 2.


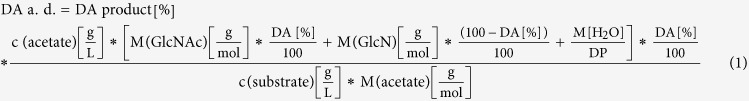



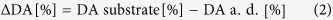


### Enzyme characterization

The standard assay involved the incubation of substrates (1 mg/ml) with PesCDA (4.0 μg/ml) in 50 mM TEA (pH 7.0) at 37 °C. The reaction was stopped by adding 1/75 volume of 1 M HCl and incubating for 5 min at 95 °C. Enzyme activity was measured based on the amount of released acetate as described above. The pH optimum for PesCDA was determined by incubating 4 μg/ml of the enzyme with 1 mg/ml chitin pentamer (Seikagaku, Tokyo, Japan) (1 mg/ml) for 2 h in three different buffers: (A) 50 mM triethanolamine (TEA), pH 7; (B) 20 mM ammonium formate, 40 mM TEA, 40 mM potassium dihydrogen phosphate, 40 mM disodium phosphate within the pH range 3–10; and (C) Teorell-Stenhagen buffer within the pH range pH 10−12. The temperature optimum was measured with the same substrate for 1 h in the temperature range 4−85 °C. The influence of different divalent cations (Ca^2+^, Fe^2+^, Mg^2+^, Mn^2+^ and, Zn^2+^) and ETDA towards on the activity of PesCDA was tested by pre-incubating the enzyme for 30 min at room temperature in 50 mM TEA buffer (pH 7.0) containing 1 mM of CaCl_2_, FeCl_2_, MgCl_2_, MnCl_2_, ZnCl_2_, or EDTA. The reaction at 37 °C was stopped 2 h after addition of the chitin pentamer (1 mg/ml) to the pre-incubated enzyme. The chitin tetramer (A_4_) (1 mg/ml) was incubated with PesCDA for 2 h at 37 °C in 50 mM TEA (pH 7.0) in 20 μl containing 0–100 mM ammonium acetate to investigate the influence of acetate on PesCDA. The reaction was stopped by adding 5 μl of 5% (v/v) formic acid, and hydrophilic-interaction liquid chromatography-electrospray ionization mass-spectrometry (HILIC-ESI-MS) was used to measure the intensity of the mono-deacetylated chitosan tetramer (A_3_D_1_) reaction product. Chitin and chitosan polymers were incubated for 24 h under the standard conditions with PesCDA.

### paCOS production for bioactivity assays

We incubated 70 μg chitin hexamer in 50 mM NH_4_HCO_3_ pH 8.0 with 0.28 μg PesCDA in a total volume of 70 μl for 2, 7, 24, and 48 h with PesCDA to generate different paCOS hexamers. The chitosan hexamer control (56 μg in 70 μl 50 mM NH_4_HCO_3_ pH 8.0), the chitin hexamer control (70 μg in 50 mM NH_4_HCO_3_ pH 8.0), the chitin hexamer with deactivated enzyme (70 μg in 50 mM NH_4_HCO_3_ pH 8.0 with inactivated enzyme (incubated for 30 min at 98 °C)) were incubated for 24 h at 37 °C. The reactions were stopped by removing the enzymes with 3 K PES filter (VWR, Darmstadt, Germany) at 4 °C, 13000 rpm for 30 min. The samples were freeze dried afterwards and dissolved in 210 μl H_2_O. The enzymatically produced chitin and chitosan oligomers were analysed by HILIC-ESI-MS.

### MALDI-TOF-MS parameters

We mixed 0.7 μl of the sample (1 mg/ml) with 0.7 μl 10 mg/ml 2,5-dihydroxybenzoic acid in a 1:1 mix of acetonitrile and water. This was placed on the target plate and dried before analysis using an Autoflex Speed mass spectrometer (Bruker, Bremen, Germany) with a SmartBeam^TM^NdYAG-laser (355 nm wavelength). For the MALDI-TOF-MS/MS experiment, we mixed 1.0 μl 3-Aminoquinoline (3-AMQ) matrix (20 mg/ml in a 2:1 mix of acetonitrile and water, pH 5.5) with 1.0 μl of the sample directly on the target plate^51^.

### HILIC-ESI-MS analyses of chitin and chitosan oligomers

HILIC-ESI-MS analysis was carried out as previously described^52^. In case of the acetate inhibitor test, we injected 1.0 μl of the samples containing 0.75 μg chitin/chitosan oligomers. The extracted ion chromatogram (EIC) of A_3_D_1_ (m/z 789.2, H^+^ adduct) was used to calculate the peak area of this chitosan oligomer. For the analysis of the enzymatically produced paCOS we injected 0.5 μl of paCOS solution containing 0.27 mM of the oligomers.

### Bioactivity assay

Oxidative burst experiments were performed using suspension-cultured *Oryza sativa* cells as described before^53^ with minor modifications. Cells were pre-incubated in L012 (Wako Chemicals, Neuss; Germany) before they were elicited manually with paCOS (final concentration 0.015 mM) or control solutions. Chemiluminescence was recorded for 0.5 s/well every minute over a period of 30 min.

### Substrates

Chitin oligomers (dimer to hexamer) were obtained from Seikagaku (Tokyo, Japan), chitosan polymers and the colloidal chitin polymer were produced and characterized as previously described^54^,^55^. The α-chitin and β-chitin polymers were kindly provided by from Mahtani Chitosan PVT. Ltd (Veraval, India).

## Additional Information

**How to cite this article**: Cord-Landwehr, S. *et al*. A chitin deacetylase from the endophytic fungus *Pestalotiopsis* sp. efficiently inactivates the elicitor activity of chitin oligomers in rice cells. *Sci. Rep.* **6**, 38018; doi: 10.1038/srep38018 (2016).

**Publisher's note:** Springer Nature remains neutral with regard to jurisdictional claims in published maps and institutional affiliations.

## Supplementary Material

Supplementary Information

## Figures and Tables

**Figure 1:**
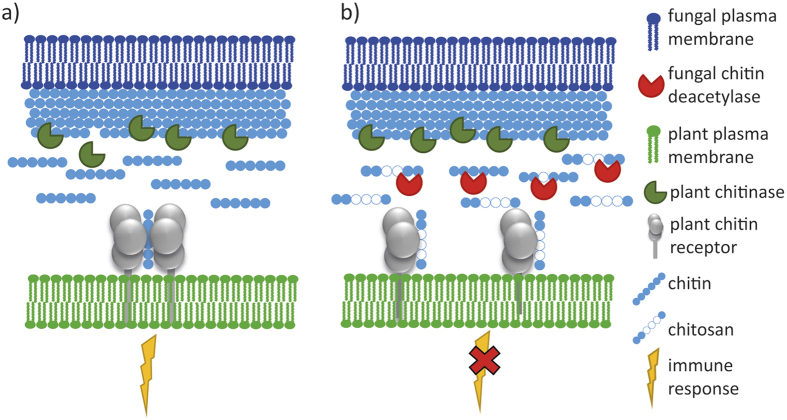
Model for plant cell recognition of fungi containing chitin in their cell walls (**a**) and hypothetical fungal strategy to overcome recognition by the plant immune system (**b**). Chitin in the fungal cell wall, consisting of *N*-acetyl-D-glucosamine units, is degraded by plant chitinases. The resulting chitin oligomers are binding to chitin-specific receptor subunits in the plant cell membrane, leading to dimerization and the triggering of immune responses. Secretion of chitin deacetylases by the fungus leads to partial deacetylation of these elicitor-active chitin oligomers into partially acetylated chitosan oligomers (paCOS). These paCOS can still bind to the monomeric receptor, preventing rather than allowing receptor dimerization, thus preventing the induction of defense reactions.

**Table 1:** Architecture of paCOS generated by incubating chitin hexamer (1 mg/ml) with PesCDA (4 μg/ml) under standard conditions for different time periods, as indicated.

sample		incubation time [h]	DA product	PA product
I	t1	2	A6	AADAAA
			**A5D1**	AAADAA
				AAAADA
II	t2	7	A5D1	AADDAA
			**A4D2**	AADADA
				AAADDA
III	t3	24	A4D2	AADDDA
			**A3D3**	
IV	t4	48	A4D2	AADDDA
			**A3D3**	
V	A6 ctrl	24	**A6**	AAAAAA
VI	D6 ctrl	24	**D6**	DDDDDD
VII	A6* ctrl	24	**A6**	AAAAAA

The controls V-VI were incubated for 24 h under the same conditions as the samples I-IV but without enzyme (V) or with heat-deactivated enzyme (VII). The most prominent peak in each sample is marked in bold. All possible patterns (see Fig. 5) of the main intermediate products at each time point are shown. A: *N-*acetyl-d-glucosamine, D: d-glucosamine.
