# Supplementary material for: A chitin deacetylase from the endophytic fungus Pestalotiopsis sp. efficiently inactivates the elicitor activity of chitin oligomers in rice cells
Source: Sci Rep. 2016 Nov 30;6:38018. doi: 10.1038/srep38018 (PMC5128826; doi:10.1038/srep38018)
Supplement: Supplementary Information [file srep38018-s1.pdf]

A chitin deacetylase from the endophytic fungus *Pestalotiopsis* sp. efficiently inactivates the elicitor activity of chitin oligomers in rice cells

Stefan Cord-Landwehr[1], Rebecca L. J. Melcher[1], Stephan Kolkenbrock[1,2] & Bruno M. Moerschbacher[1*]

[1] Institut für Biologie und Biotechnologie der Pflanzen, Westfälische Wilhelms-Universität Münster, Schlossplatz 8, 48143 Münster, Germany

[2] current address: altona Diagnostics GmbH, Hamburg, Germany

* corresponding author

moersch@uni-muenster.de

Supplementary figures

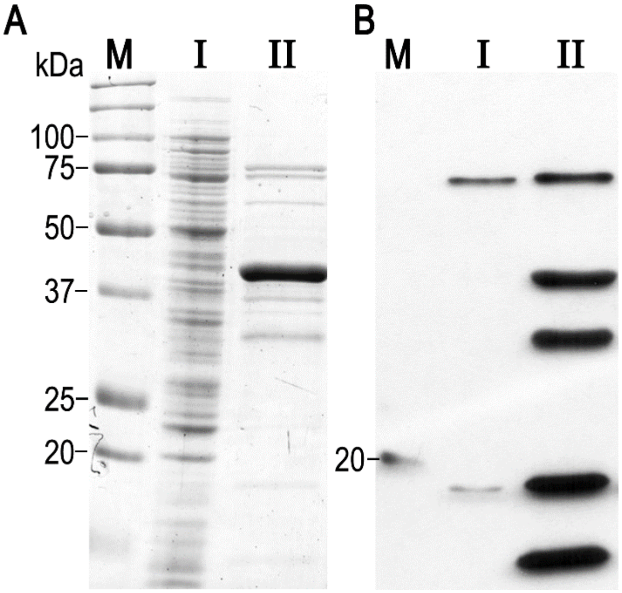

Supplementary Figure S1 SDS-PAGE (A) and western blot analysis (B) of the crude extract of *E. coli* Rosetta2 (DE3) [pLysSRARE2] [pET22b::NSt-MBP-CDA-CSt] cells and the streptactin purified protein. We used 30 µg of the crude extract (I) and 3 µg purified protein (II) for both methods. A HRP-coupled streptactin conjugate was used in the enhanced chemiluminescence western blot. M: Precision Plus Protein™ Standards All Blue (Bio-Rad, Hercules, CA, USA).

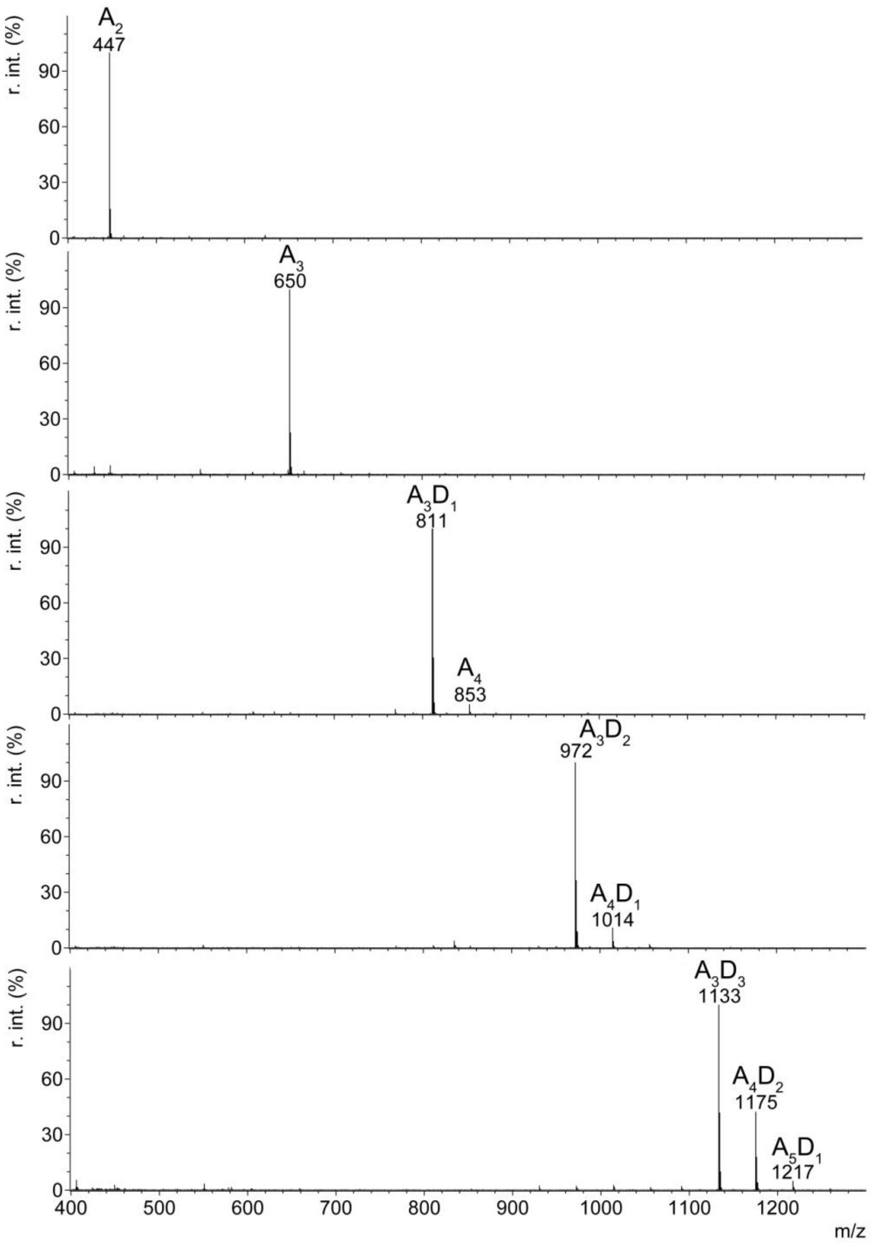

Supplementary Figure S2. MALDI-TOF-MS analysis of chitin oligomers (DP 2–6) incubated with PesCDA. The chitin oligomers (1 mg/ml) were incubated for 24 h with PesCDA (4 µg/ml) under the standard conditions. We used 10 mg/ml 2,5-dihydroxybenzoic acid in 2:1 acetonitrile:water as the matrix. All labelled peaks are single charged ions of chitin or chitosan oligomers as sodium adducts. A: *N*-acetyl-D-glucosamine; D: D-glucosamine

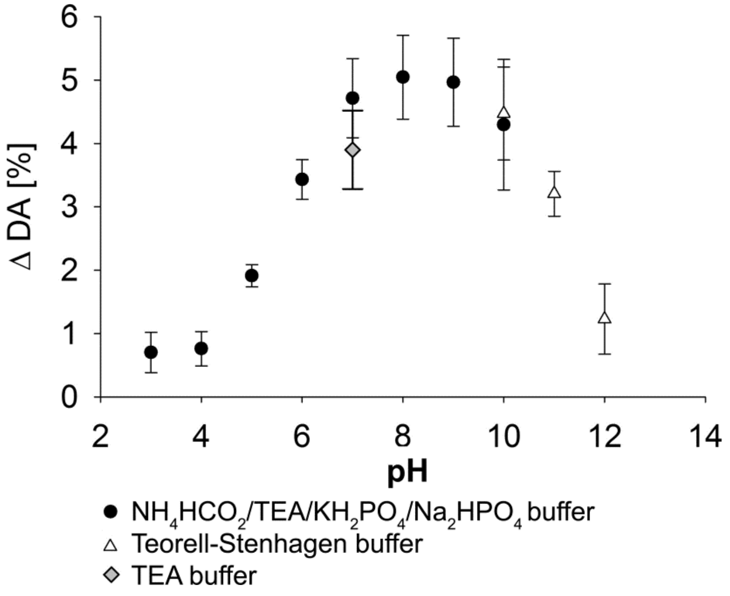

Supplementary Figure S3. The pH optimum of PesCDA. Chitin pentamer (1 mg/ml) was incubated for 2 h at 37°C with PesCDA (4 µg/ml) in three different buffers: (A) 50 mM TEA pH 7.0; (B) 20 mM ammonium formate, 40 mM TEA, 40 mM KH_2PO_4 and 40 mM Na_2HPO_4 within the pH range 3–10; and (C) Teorell-Stenhagen buffer within the pH range 10–12. The activity of the enzyme (ΔDA) was calculated based on the amount of released acetate. The standard deviation was calculated based on three independent experiments.

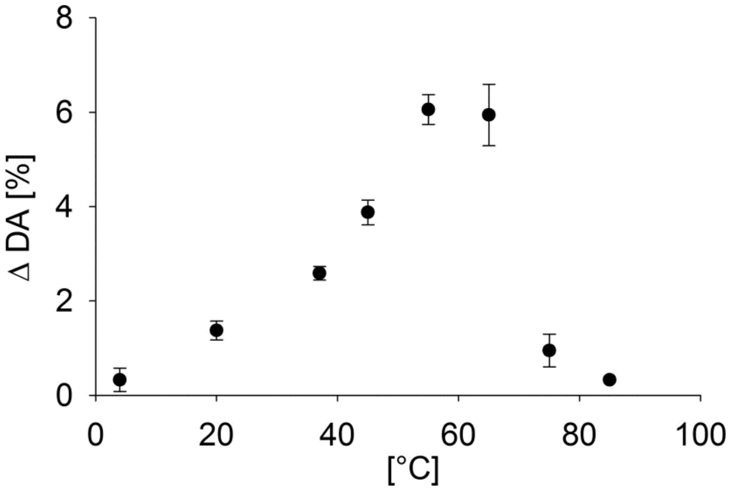

Supplementary Figure S4. The temperature optimum of PesCDA. Chitin pentamer (1 mg/ml) was incubated for 1 h with PesCDA (4 µg/ml) in 50 mM TEA (pH 7.0) in the temperature range 4–85°C. The activity of the enzyme (ΔDA) was calculated based on the amount of released acetate. The standard deviation was calculated based on three independent experiments.

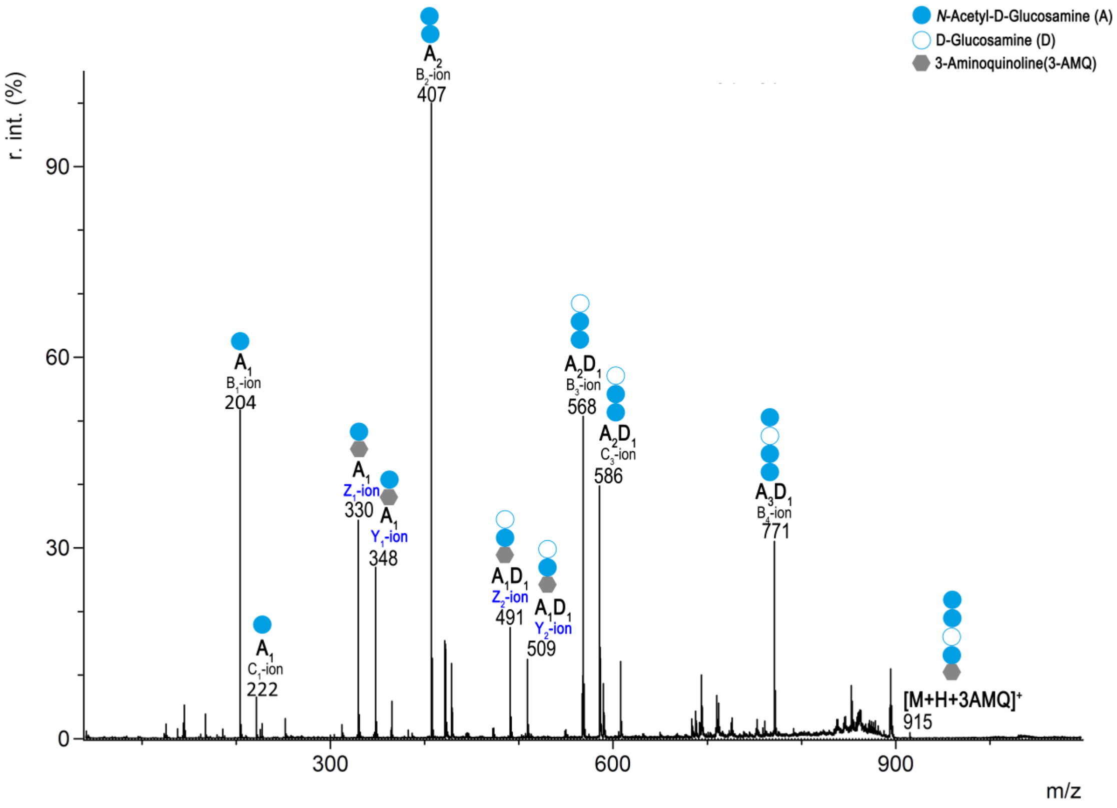

Supplementary Figure S5 MALDI-TOF-MS/MS fragmentation pattern analysis of the single-deacetylated chitin tetramer A_3D_1 (AADA) PesCDA product labelled with 3-Aminoquinoline (3-AMQ) on the reducing end as H^+ adduct.

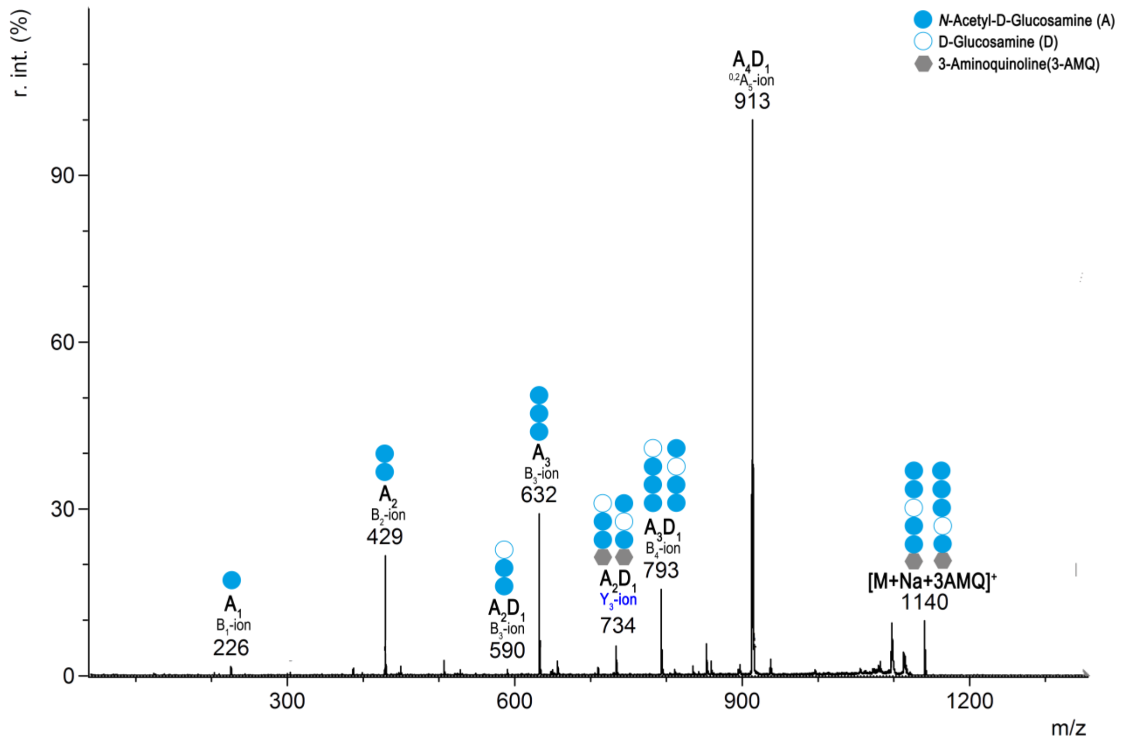

Supplementary Figure S6: MALDI-TOF-MS/MS fragmentation pattern analysis of the single-deacetylated chitin pentamer A_4D_1 (AADAA - AAADA) PesCDA product labelled with 3-Aminoquinoline (3-AMQ) on the reducing end as Na^+ adduct.

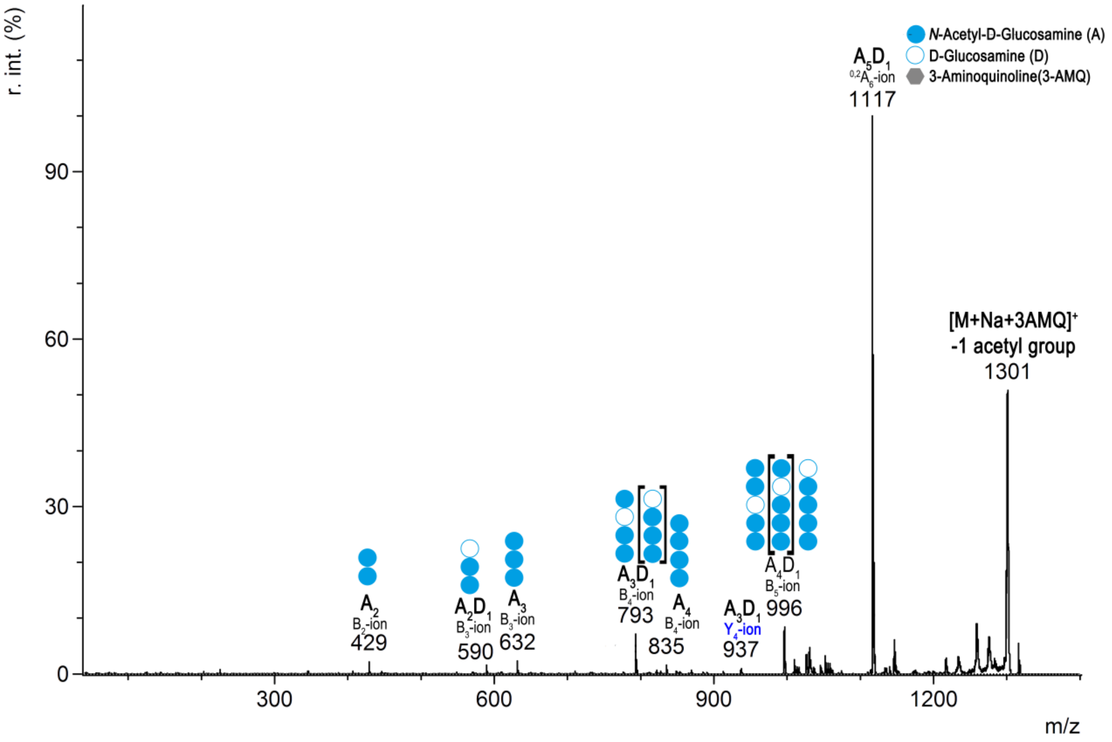

Supplementary Figure S7: MALDI-TOF-MS/MS fragmentation pattern analysis of the single-deacetylated chitin hexamer A_5D_1 (AADAAA – [AAADAA] – AAAADA) PesCDA product labelled with 3-Aminoquinoline (3-AMQ) on the reducing end as Na^+ adduct. Due to the limitations of MALDI-TOF-MS/MS analysis, it is not possible to determine unequivocally the presence of the two hexamer products given in square brackets.

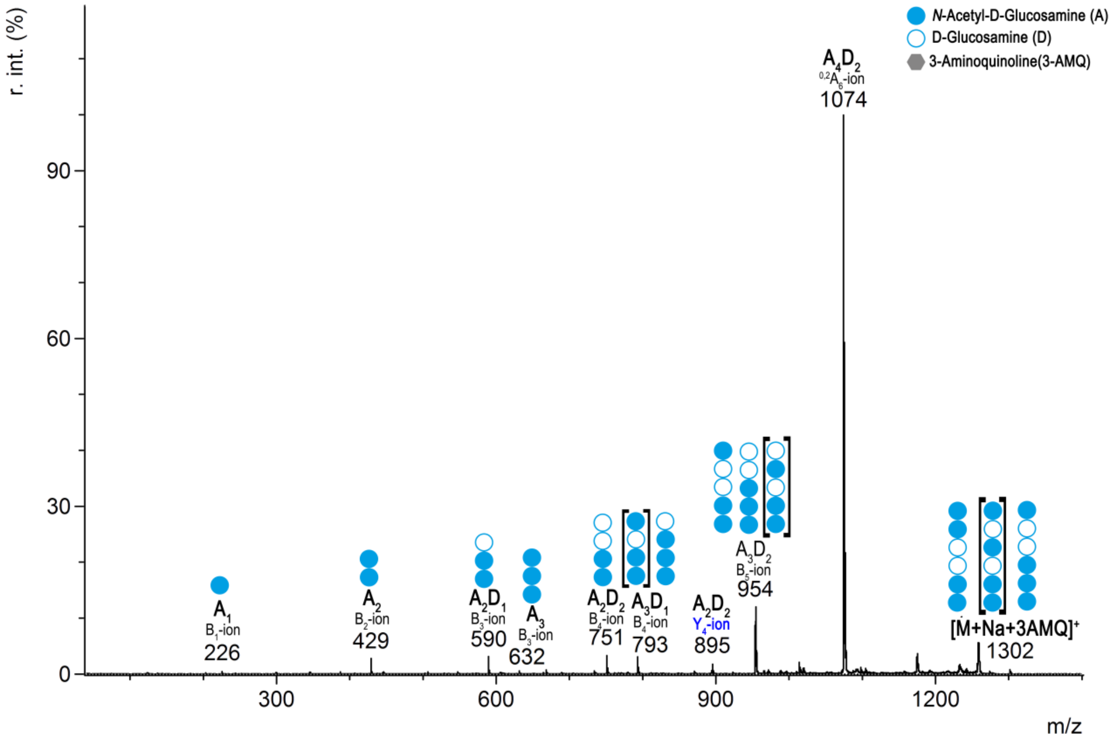

Supplementary Figure S8: MALDI-TOF-MS/MS fragmentation pattern analysis of double-deacetylated chitin hexamer A_4D_2 (AADDAA - [AADADA] - AAADDA) PesCDA products labelled with 3-Aminoquinoline (3-AMQ) on the reducing end as Na^+ adduct. Due to the limitations of MALDI-TOF-MS/MS analysis, it is not possible to determine unequivocally the presence of the two hexamer products given in square brackets.

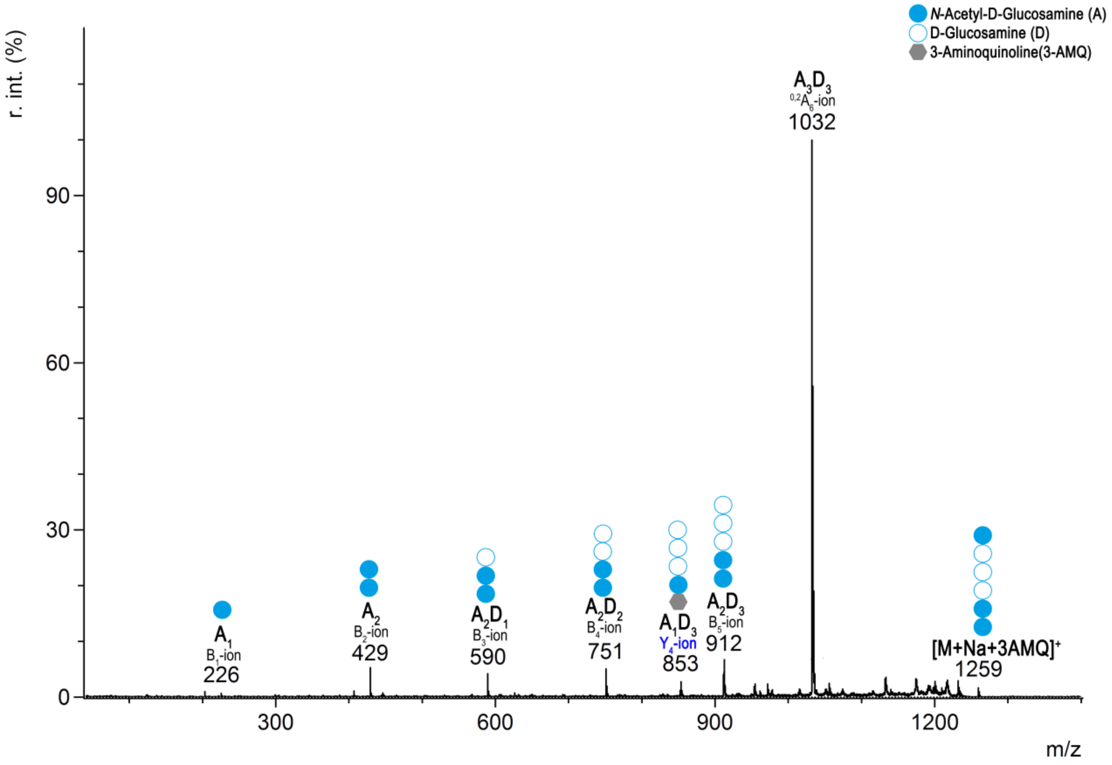

Supplementary Figure S9: MALDI-TOF-MS/MS fragmentation pattern analysis of the triple-deacetylated chitin hexamer A_3D_3 (AADDDA) labelled with 3-Aminoquinoline (3-AMQ) on the reducing end as Na^+ adduct.

Supplementary Tables

Supplementary Table 1. Influence of divalent cations on the activity of PesCDA against a chitin pentamer substrate. The enzyme was pre-incubated with the chloride salts of different divalent cations as cofactors or with EDTA for 30 min at room temperature. The activities of the enzyme (ΔDA) in three independent experiments were calculated based on the amount of acetate released during 2-h incubation at 37°C in 50 mM TEA (pH 7.0). The standard deviation was calculated based on three independent experiments.

**divalent cation (1 mM)**	**ΔDA**
native protein	7.97 ± 0.63
Ca^{2+}	7.64 ± 0.71
Fe^{2+}	0.33 ± 0.39
Mg^{2+}	7.95 ± 0.55
Mn^{2+}	3.89 ± 0.31
Zn^{2+}	6.23 ± 0.70
EDTA	7.27 ± 0.56

Supplementary Table 2. Effect of acetate on the activity of PesCDA against a chitin tetramer substrate. The chitin tetramer (A_4) (1 mg/ml) was incubated with PesCDA for 2 h at 37°C in 50 mM TEA (pH 7.0) containing 0-100 mM ammonium acetate. The reaction was stopped by adding formic acid and hydrophilic-interaction liquid chromatography-electrospray ionization mass-spectrometry (HILIC-ESI-MS) was used to measure the intensity of the mono-deacetylated chitosan tetramer (A_3D_1) reaction product. The extracted ion chromatogram of the mono-deacetylated chitosan tetramer (m/z 789.3) was used to determine the peak area. The standard deviation was calculated based on three independent experiments.

**c ammonium acetate [mM]**	**EIC peak area A_3D_1 (*m/z* 789.32)**	**activity (norm.)**
0	626477205	100.0 ± 7.0
5	618293504	98.7 ± 8.0
25	589206741	94.1 ± 4.9
50	534792458	85.4 ± 2.6
75	502763296	80.3 ± 7.9
100	495564544	79.1 ± 2.6
